# Supplementary material for: Multi-qubit quantum computing using discrete-time quantum walks on closed graphs
Source: Sci Rep. 2023 Jul 26;13:12078. doi: 10.1038/s41598-023-39061-1 (PMC10372037; doi:10.1038/s41598-023-39061-1)
Supplement: Supplementary file 1 — Supplementary Information. [file 41598_2023_39061_MOESM1_ESM.pdf]

Supplementary Information

S1 An alternative approach to scaling the DTQW to N qubits

The scheme presented for universal quantum computation using quantum walk for three qubit equivalent system as shown in [1] can also be scaled to larger qubit system. It is done by using the position space of the preceding sets of the quantum walk system as the coin for the next set of quantum walk. Using the preceding set of walk as coin implies that the quantum walk is conditioned on the output of the preceding set of walk. Below we will show the scalability for four- and five-qubit systems and then extend it to N-qubit system.
Form of shift operators which is used through out for scaling of the universal computation model as given in Ref. [1] for input state $|k, m, p\rangle$ where, $|k\rangle$ is the coin state with two degree of freedom, $|m\rangle$ and $|p\rangle$ are the position states of two different cyclic quantum walk with four state $span\{0, 1, 2, 3\}$, respectively, is,

$$\hat{W}^0_\pm \equiv \big(\hat{\sigma_x} \otimes |m\rangle\langle m| + \sum_{n\neq m} \mathbb{1}_2 \otimes |n\rangle\langle n|\big)\hat{S}^k_{2,\pm}\big(\hat{\sigma_x} \otimes \mathbb{1}\big),$$
$$\hat{W}^1_\pm \equiv \big(\hat{\sigma_x} \otimes |m\rangle\langle m| + \sum_{n\neq m} \mathbb{1}_2 \otimes |n\rangle\langle n|\big)\hat{S}^k_{2,\pm}\big(\hat{\sigma_z} \otimes \mathbb{1}\big), \tag{1}$$

and

$$\hat{V}^0_\pm \equiv \big(\hat{\sigma_{x2}} \otimes |p\rangle\langle p| + \sum_{q\neq p} \mathbb{1}_4 \otimes |q\rangle\langle q|\big)\hat{S}^m_{4,\pm}\big(\hat{\sigma_{x2}} \otimes \mathbb{1}\big) \equiv \hat{V}^3_\pm$$
$$\hat{V}^1_\pm \equiv \big(\hat{\sigma_{x2}} \otimes |p\rangle\langle p| + \sum_{q\neq p} \mathbb{1}_4 \otimes |q\rangle\langle q|\big)\hat{S}^m_{4,\pm}\big(\hat{\sigma_{z2}} \otimes \mathbb{1}\big) \equiv \hat{V}^2_\pm, \tag{2}$$

where, $\hat{\sigma_{x2}} = \mathbb{1}_2 \otimes \hat{\sigma_x}$ and on quantum walk system it is given as,

$$\mathbb{1}_2 \otimes \hat{\sigma_{x2}}|k, 00\rangle = \hat{S}^0_{2,+}\hat{S}^1_{2,+}|k, 0\rangle$$
$$\mathbb{1}_2 \otimes \hat{\sigma_{x2}}|k, 01\rangle = \hat{S}^0_{2,-}\hat{S}^1_{2,-}|k, 1\rangle$$
$$\mathbb{1}_2 \otimes \hat{\sigma_{x2}}|k, 11\rangle = \hat{S}^0_{2,+}\hat{S}^1_{2,+}|k, 2\rangle$$
$$\mathbb{1}_2 \otimes \hat{\sigma_{x2}}|k, 10\rangle = \hat{S}^0_{2,-}\hat{S}^1_{2,-}|k, 3\rangle. \tag{3}$$

If the coin state is 2-qubit equivalent then $\hat{\sigma_{x2}} = \mathbb{1}_2 \otimes \hat{\sigma_x}$ on quantum walk system it is given as,

$$\mathbb{1}_4 \otimes \hat{\sigma_{x2}}|m, 00\rangle = \hat{S}^0_{4,+}\hat{S}^1_{4,+}\hat{S}^2_{4,+}\hat{S}^3_{4,+}|m, 0\rangle$$
$$\mathbb{1}_4 \otimes \hat{\sigma_{x2}}|m, 01\rangle = \hat{S}^0_{4,-}\hat{S}^1_{4,-}\hat{S}^2_{4,+}\hat{S}^3_{4,+}|m, 1\rangle$$
$$\mathbb{1}_4 \otimes \hat{\sigma_{x2}}|m, 11\rangle = \hat{S}^0_{4,+}\hat{S}^1_{4,+}\hat{S}^2_{4,+}\hat{S}^3_{4,+}|m, 2\rangle$$
$$\mathbb{1}_4 \otimes \hat{\sigma_{x2}}|m, 10\rangle = \hat{S}^0_{4,-}\hat{S}^1_{4,-}\hat{S}^2_{4,+}\hat{S}^3_{4,+}|m, 3\rangle \tag{4}$$

and $\hat{\sigma_{z2}} = \mathbb{1}_2 \otimes \hat{\sigma_z}$ and its action on a quantum walk system is defined by,

$$\hat{\sigma_{z2}}|00\rangle = \mathbb{1}_2|m = 0\rangle$$
$$\hat{\sigma_{z2}}|01\rangle = -\mathbb{1}_2|m = 1\rangle$$
$$\hat{\sigma_{z2}}|11\rangle = -\mathbb{1}_2|m = 2\rangle$$
$$\hat{\sigma_{z2}}|10\rangle = \mathbb{1}_2|m = 3\rangle \tag{5}$$

The total number of states for this case will be equivalent to combined state of the Hilbert-space $\mathcal{H}_c \otimes \mathcal{H}_{p1} \otimes \mathcal{H}_{p2}$.

$$
\begin{aligned}
\hat{H}_4 \left|j\right\rangle \otimes \left|00\right\rangle \otimes \left|0\right\rangle &\rightarrow (\mathbb{1}_2 \otimes \hat{V}_+^0)(\mathbb{H}_3 \otimes \mathbb{1}_2)(\left|j, m=0, 0\right\rangle) \\
&= (\mathbb{1}_2 \otimes \big(\hat{\sigma_{x2}} \otimes \left|0\right\rangle\left\langle 0\right| + \mathbb{1}_4 \otimes \left|1\right\rangle\left\langle 1\right|\big)\hat{S}^0_{4,+}\big(\hat{\sigma_{x2}} \otimes \mathbb{1}\big))(\hat{W}_+^{(j \mod 2)} \otimes \mathbb{1})(\hat{H}_1 \otimes \mathbb{1} \otimes \mathbb{1})(\left|j, m=0, 0\right\rangle) \\
&= (\mathbb{1}_2 \otimes \big(\hat{\sigma_{x2}} \otimes \left|0\right\rangle\left\langle 0\right| + \mathbb{1}_4 \otimes \left|1\right\rangle\left\langle 1\right|\big)\hat{S}^0_{4,+}\big(\hat{\sigma_{x2}} \otimes \mathbb{1}\big))\frac{1}{\sqrt{2}}(\left|j, m=0, 0\right\rangle + \left|j, m=1, 0\right\rangle) \\
&= (\mathbb{1}_2 \otimes \big(\hat{\sigma_{x2}} \otimes \left|0\right\rangle\left\langle 0\right| + \mathbb{1}_4 \otimes \left|1\right\rangle\left\langle 1\right|\big)\hat{S}^0_{4,+})\frac{1}{\sqrt{2}}(\left|j, m=1, 0\right\rangle + \left|j, m=0, 0\right\rangle) \\
&= (\mathbb{1}_2 \otimes \big(\hat{\sigma_{x2}} \otimes \left|0\right\rangle\left\langle 0\right| + \mathbb{1}_4 \otimes \left|1\right\rangle\left\langle 1\right|\big))\frac{1}{\sqrt{2}}(\left|j, m=1, 0\right\rangle + \left|j, m=0, 1\right\rangle) \\
&= \frac{1}{\sqrt{2}}(\left|j, m=0, 0\right\rangle + \left|j, m=0, 1\right\rangle) \\
&= \left|j\right\rangle \otimes \left|00\right\rangle \otimes \frac{\big(\left|0\right\rangle + \left|1\right\rangle\big)}{\sqrt{2}} \qquad (6)
\end{aligned}
$$

S1.1 Implementing Hadamard, phase, and CNOT-gate on four qubit equivalent system

Hadamard operation: To map the Hadamard operation on fourth qubit of the four qubit system, one can use a three-qubit equivalent quantum walk system and a set of position space with two states such that the operation is defined on $\mathcal{H}_c \otimes \mathcal{H}_4 \otimes \mathcal{H}_2$ combined Hilbert space. The three-qubit equivalent quantum walk system defined on $\mathcal{H}_c \otimes \mathcal{H}_4$ will act as a coin for the next set of position space with two states $span\{\left|0\right\rangle, \left|1\right\rangle\}$ defined on $\mathcal{H}_2$. One can realize the Hadamard operation on the fourth qubit of the four-qubit system by using a combination of coin and shift operators as,

$$
\begin{aligned}
\hat{H}_4 \left|j, m, 0\right\rangle &\rightarrow (\mathbb{1}_2 \otimes \hat{V}_+^{(m \mod 4)})(\hat{H}_3 \otimes \mathbb{1}_2)\left|j, m, 0\right\rangle, \\
\hat{H}_4 \left|j, m, 1\right\rangle &\rightarrow (\mathbb{1}_2 \otimes \hat{V}_-^{(m+1 \mod 4)})(\hat{H}_3 \otimes \mathbb{1}_2)\left|j, m, 1\right\rangle \qquad (7)
\end{aligned}
$$

where, $\hat{H}_3$ is given by the quantum walk scheme presented in the Ref. [1]. $\left|j\right\rangle$ is the basis state of coin Hilbert space such that $\left|j\right\rangle = \{\left|0\right\rangle, \left|1\right\rangle\}$, $\left|l\right\rangle$ represent two qubit equivalent cyclic position Hilbert state $\mathcal{H}_4$ given by, $\left|m\right\rangle = \{\left|x=0\right\rangle, \left|x=1\right\rangle, \left|x=2\right\rangle, \left|x=3\right\rangle\} \equiv \{\left|00\right\rangle, \left|01\right\rangle, \left|11\right\rangle, \left|10\right\rangle\}$. $\hat{H}_4 \equiv \mathbb{1} \otimes \mathbb{1} \otimes \mathbb{1} \otimes \hat{H}$ and an illustration of this scaling when $\left|m=0\right\rangle$ is given in Eq. (6). *Phase operation:* To map the phase gate operation on fourth qubit of the four qubit system, one can again use a three-qubit equivalent quantum walk system as coin for the position space with two states $span\{\left|0\right\rangle, \left|1\right\rangle\}$. The walk is again defined on the Hilbert space $\mathcal{H}_c \otimes \mathcal{H}_4 \otimes \mathcal{H}_2$. The operations that will evolve the initial state of the quantum walk into the state with phase on the fourth qubit is,

$$
\begin{aligned}
\hat{P}_4 \left|j, m, 0\right\rangle &\rightarrow \mathbb{1}_2 \otimes \mathbb{1}_4 \otimes \mathbb{1}_2 \left|j, m, 0\right\rangle \\
\hat{P}_4 \left|j, m, 1\right\rangle &\rightarrow \hat{\Phi} \otimes \mathbb{1}_4 \otimes \mathbb{1}_2 \left|j, m, 1\right\rangle \qquad (8)
\end{aligned}
$$

where, $\hat{\Phi} = e^{i\phi}\mathbb{1}_2$ and $\hat{P}_4 \equiv \mathbb{1} \otimes \mathbb{1} \otimes \mathbb{1} \otimes \hat{P}$. *Controlled-NOT operation:* To map the CNOT gate operation when the fourth qubit is the target and other qubits are control of the four qubit system, we will again need a three-qubit equivalent quantum walk system as coin for the next set of position space $span\{\left|0\right\rangle, \left|1\right\rangle\}$. The quantum walk scheme when fourth qubit is the target for various control qubit $CNOT_{i4}$, where i is the control qubit, is given by,

$$
\begin{aligned}
C\hat{N}OT_{14} \left|j, m, 0\right\rangle &\rightarrow (\hat{S}^0_{2,-} \otimes \mathbb{1}_2)(\mathbb{1}_2 \otimes \hat{S}^m_{4,+})(\hat{S}^0_{2,+} \otimes \mathbb{1}_2) \\
C\hat{N}OT_{14} \left|j, m, 1\right\rangle &\rightarrow (\hat{S}^0_{2,-} \otimes \mathbb{1}_2)(\mathbb{1}_2 \otimes \hat{S}^m_{4,-})(\hat{S}^0_{2,+} \otimes \mathbb{1}_2); \qquad (9)
\end{aligned}
$$

$$
\begin{aligned}
C\hat{N}OT_{24} \left|j, m, 0\right\rangle &\rightarrow (\mathbb{1}_2 \otimes \hat{S}^3_{4,+})(\mathbb{1}_2 \otimes \hat{S}^2_{4,+}) \\
C\hat{N}OT_{24} \left|j, m, 1\right\rangle &\rightarrow (\mathbb{1}_2 \otimes \hat{S}^3_{4,-})(\mathbb{1}_2 \otimes \hat{S}^2_{4,-}) \qquad (10)
\end{aligned}
$$

and

$$
\begin{aligned}
C\hat{N}OT_{34} \left|j, m, 0\right\rangle &\rightarrow (\mathbb{1}_2 \otimes \hat{S}^1_{4,+})(\mathbb{1}_2 \otimes \hat{S}^2_{4,+}) \\
C\hat{N}OT_{34} \left|j, m, 1\right\rangle &\rightarrow (\mathbb{1}_2 \otimes \hat{S}^1_{4,-})(\mathbb{1}_2 \otimes \hat{S}^2_{4,-}) \qquad (11)
\end{aligned}
$$

The quantum walk scheme when fourth qubit is the control for various target qubit $CNOT_{4i}$, where i is the target qubit and is given by,

$$
\begin{aligned}
C\hat{N}OT_{41} \left|j, m, 0\right\rangle &\rightarrow \mathbb{1} \\
C\hat{N}OT_{41} \left|j, m, 1\right\rangle &\rightarrow (\hat{\sigma}_x \otimes \mathbb{1}_4 \otimes \mathbb{1}_2); \qquad (12)
\end{aligned}
$$

$$\hat{CNOT}_{42}\,|j,m,0\rangle \rightarrow \mathbb{1}$$
$$\hat{CNOT}_{42}\,|j,m,1\rangle \rightarrow (\mathbb{1}_2 \otimes \hat{\sigma}_{x1}) \tag{13}$$

where, $\hat{\sigma_{x1}} = \hat{\sigma_x} \otimes \mathbb{1}_2$ and on quantum walk system it is given as,

$$\begin{aligned}
\mathbb{1}_2 \otimes \hat{\sigma_{x1}}\,|k,00\rangle &= \hat{S}^0_{2,-}\hat{S}^1_{2,-}\,|k,0\rangle \\
\mathbb{1}_2 \otimes \hat{\sigma_{x1}}\,|k,01\rangle &= \hat{S}^0_{2,+}\hat{S}^1_{2,+}\,|k,1\rangle \\
\mathbb{1}_2 \otimes \hat{\sigma_{x1}}\,|k,11\rangle &= \hat{S}^0_{2,-}\hat{S}^1_{2,-}\,|k,2\rangle \\
\mathbb{1}_2 \otimes \hat{\sigma_{x1}}\,|k,10\rangle &= \hat{S}^0_{2,+}\hat{S}^1_{2,+}\,|k,3\rangle
\end{aligned} \tag{14}$$

and

$$\hat{CNOT}_{43}\,|j,m,0\rangle \rightarrow \mathbb{1}$$
$$\hat{CNOT}_{43}\,|j,m,1\rangle \rightarrow (\mathbb{1}_2 \otimes \hat{\sigma}_{x2}) \tag{15}$$

where, $\hat{\sigma_{x2}} = \mathbb{1}_2 \otimes \hat{\sigma_x}$ on quantum walk system is given by Eq. (3).

S1.2 Implementing Hadamard, phase, and Controlled-NOT operation on five qubit equivalent system

Hadamard Operation: The Hadamard operation on fourth qubit of the five qubit system is defined on two cyclic quantum walk system with four position states. The combined Hilbert space is $\mathcal{H}_c \otimes \mathcal{H}_4 \otimes \mathcal{H}_4$. Position state of the previous quantum walk system will act as a coin for the position space of the next quantum walk system. One can realize the Hadamard operation on the fourth qubit of the five-qubit system by using a combination of coin and shift operators as,

$$\begin{aligned}
\hat{H}_4\,|j,m,00\rangle &\rightarrow (\mathbb{1}_2 \otimes \hat{V}_-^{(m \mod 4)})(\hat{H}_3 \otimes \mathbb{1}_4)\,|j,m,p=0\rangle\,, \\
\hat{H}_4\,|j,m,01\rangle &\rightarrow (\mathbb{1}_2 \otimes \hat{V}_+^{(m \mod 4)})(\hat{H}_3 \otimes \mathbb{1}_4)\,|j,m,p=1\rangle\,, \\
\hat{H}_4\,|j,m,11\rangle &\rightarrow (\mathbb{1}_2 \otimes \hat{V}_-^{(m+1 \mod 4)})(\hat{H}_3 \otimes \mathbb{1}_4)\,|j,m,p=2\rangle\,, \\
\hat{H}_4\,|j,m,10\rangle &\rightarrow (\mathbb{1}_2 \otimes \hat{V}_+^{(m+1 \mod 4)})(\hat{H}_3 \otimes \mathbb{1}_4)\,|j,m,p=3\rangle
\end{aligned} \tag{16}$$

and

$$\begin{aligned}
\hat{H}_5\,|j,m,00\rangle &\rightarrow (\mathbb{1}_2 \otimes \hat{V}_+^{(m \mod 4)})(\hat{H}_3 \otimes \mathbb{1}_4)\,|j,m,p=0\rangle\,, \\
\hat{H}_5\,|j,m,01\rangle &\rightarrow (\mathbb{1}_2 \otimes \hat{V}_-^{(m+1 \mod 4)})(\hat{H}_3 \otimes \mathbb{1}_4)\,|j,m,p=1\rangle\,, \\
\hat{H}_5\,|j,m,11\rangle &\rightarrow (\mathbb{1}_2 \otimes \hat{V}_+^{(m+1 \mod 4)})(\hat{H}_3 \otimes \mathbb{1}_4)\,|j,m,p=2\rangle\,, \\
\hat{H}_5\,|j,m,10\rangle &\rightarrow (\mathbb{1}_2 \otimes \hat{V}_-^{(m \mod 4)})(\hat{H}_3 \otimes \mathbb{1}_4)\,|j,m,p=3\rangle\,.
\end{aligned} \tag{17}$$

Here $\hat{H}_3$ is again given in the Ref. [1], $\hat{H}_4 \equiv \mathbb{1} \otimes \mathbb{1} \otimes \mathbb{1} \otimes \hat{H} \otimes \mathbb{1}$ and $\hat{H}_5 \equiv \mathbb{1} \otimes \mathbb{1} \otimes \mathbb{1} \otimes \mathbb{1} \otimes \hat{H}$. *Phase operation:* Similarly, the quantum walk scheme for the phase operation on fourth qubit of the five qubit system is also defined on the Hilbert space $\mathcal{H}_c \otimes \mathcal{H}_4 \otimes \mathcal{H}_4$. The operations that will evolve the initial state of the quantum walk into the state with phase on the fourth qubit is,

$$\begin{aligned}
\hat{P}_4\,|j,m,00\rangle &\rightarrow \mathbb{1}\,|j,m,p=0\rangle\,, \\
\hat{P}_4\,|j,m,01\rangle &\rightarrow \mathbb{1}\,|j,m,p=1\rangle\,, \\
\hat{P}_4\,|j,m,11\rangle &\rightarrow (\hat{\Phi} \otimes \mathbb{1})\,|j,m,p=2\rangle\,, \\
\hat{P}_4\,|j,m,10\rangle &\rightarrow (\hat{\Phi} \otimes \mathbb{1})\,|j,m,p=3\rangle\,.
\end{aligned} \tag{18}$$

and phase operation on the fifth qubit is,

$$\begin{aligned}
\hat{P}_5\,|j,m,00\rangle &\rightarrow \mathbb{1}\,|j,m,p=0\rangle\,, \\
\hat{P}_5\,|j,m,01\rangle &\rightarrow (\hat{\Phi} \otimes \mathbb{1})\,|j,m,p=1\rangle\,, \\
\hat{P}_5\,|j,m,11\rangle &\rightarrow (\hat{\Phi} \otimes \mathbb{1})\,|j,m,p=2\rangle\,, \\
\hat{P}_5\,|j,m,10\rangle &\rightarrow \mathbb{1}\,|j,m,p=3\rangle\,.
\end{aligned} \tag{19}$$

Table 1. Hadamard operation $\hat{H}$ on even $(n-1)^{\text{th}}$ and odd n^{th} qubit when the processor has n number of qubits. $|m\rangle$ is the position basis state of the previous set of cyclic quantum walk. $|(n-1)\rangle \otimes |n\rangle$ is $\{|00\rangle, |01\rangle, |11\rangle, |10\rangle\}$ which is also equivalent to the four computational position basis state of the cyclic quantum walk.

	$\hat{H}_{(n-1)}$	$\hat{H}_n$
$\|j,...,m,00\rangle$	$(\mathbb{1}\otimes\hat{V}_-^{(m \mod 4)})(\hat{H}_{n-2}\otimes\mathbb{1}_4)\|j,...,m,p=0\rangle$	$(\mathbb{1}\otimes\hat{V}_+^{(m \mod 4)})(\hat{H}_{n-2}\otimes\mathbb{1}_4)\|j,...,m,p=0\rangle$
$\|j,...,m,01\rangle$	$(\mathbb{1}\otimes\hat{V}_+^{(m \mod 4)})(\hat{H}_{n-2}\otimes\mathbb{1}_4)\|j,...,m,p=1\rangle$	$(\mathbb{1}\otimes\hat{V}_-^{(m+1 \mod 4)})(\hat{H}_{n-2}\otimes\mathbb{1}_4)\|j,...,m,p=1\rangle$
$\|j,...,m,11\rangle$	$(\mathbb{1}\otimes\hat{V}_-^{(m+1 \mod 4)})(\hat{H}_{n-2}\otimes\mathbb{1}_4)\|j,...,m,p=2\rangle$	$(\mathbb{1}\otimes\hat{V}_+^{(m+1 \mod 4)})(\hat{H}_{n-2}\otimes\mathbb{1}_4)\|j,...,m,p=2\rangle$
$\|j,...,m,10\rangle$	$(\mathbb{1}\otimes\hat{V}_+^{(m+1 \mod 4)})(\hat{H}_{n-2}\otimes\mathbb{1}_4)\|j,...,m,p=3\rangle$	$(\mathbb{1}\otimes\hat{V}_-^{(m \mod 4)})(\hat{H}_{n-2}\otimes\mathbb{1}_4)\|j,...,m,p=3\rangle$

where, $\hat{\Phi} = e^{i\phi}\mathbb{1}_2$, $\hat{P}_4 \equiv \mathbb{1}\otimes\mathbb{1}\otimes\mathbb{1}\otimes\hat{P}\otimes\mathbb{1}$ and $\hat{P}_5 \equiv \mathbb{1}\otimes\mathbb{1}\otimes\mathbb{1}\otimes\mathbb{1}\otimes\hat{P}$.

Controlled-NOT operation: To implement the CNOT operation when the fourth and fifth qubits are the target of the five qubit system, we will need a cyclic quantum walk with four position basis state as a coin for the position space of another quantum walk on the first system with four position basis states. The quantum walk scheme when fourth qubit is the target for various control qubit $CNOT_{i4}$, where i is the control qubits, is given by,

$$\begin{aligned}
C\hat{N}OT_{14}|j,m,00\rangle &\to (\hat{S}^0_{2,-}\otimes\mathbb{1}_2)(\mathbb{1}_2\otimes\hat{S}^m_{4,-})(\hat{S}^0_{2,+}\otimes\mathbb{1}_2)\\
C\hat{N}OT_{14}|j,m,01\rangle &\to (\hat{S}^0_{2,-}\otimes\mathbb{1}_2)(\mathbb{1}_2\otimes\hat{S}^m_{4,+})(\hat{S}^0_{2,+}\otimes\mathbb{1}_2)\\
C\hat{N}OT_{14}|j,m,11\rangle &\to (\hat{S}^0_{2,-}\otimes\mathbb{1}_2)(\mathbb{1}_2\otimes\hat{S}^m_{4,-})(\hat{S}^0_{2,+}\otimes\mathbb{1}_2)\\
C\hat{N}OT_{14}|j,m,10\rangle &\to (\hat{S}^0_{2,-}\otimes\mathbb{1}_2)(\mathbb{1}_2\otimes\hat{S}^m_{4,+})(\hat{S}^0_{2,+}\otimes\mathbb{1}_2);
\end{aligned} \tag{20}$$

$$\begin{aligned}
C\hat{N}OT_{24}|j,m,00\rangle &\to (\mathbb{1}_2\otimes\hat{S}^3_{4,-})(\mathbb{1}_2\otimes\hat{S}^2_{4,-})\\
C\hat{N}OT_{24}|j,m,01\rangle &\to (\mathbb{1}_2\otimes\hat{S}^3_{4,+})(\mathbb{1}_2\otimes\hat{S}^2_{4,+})\\
C\hat{N}OT_{24}|j,m,11\rangle &\to (\mathbb{1}_2\otimes\hat{S}^3_{4,-})(\mathbb{1}_2\otimes\hat{S}^2_{4,-})\\
C\hat{N}OT_{24}|j,m,10\rangle &\to (\mathbb{1}_2\otimes\hat{S}^3_{4,+})(\mathbb{1}_2\otimes\hat{S}^2_{4,+});
\end{aligned} \tag{21}$$

$$\begin{aligned}
C\hat{N}OT_{34}|j,m,00\rangle &\to (\mathbb{1}_2\otimes\hat{S}^1_{4,-})(\mathbb{1}_2\otimes\hat{S}^2_{4,-})\\
C\hat{N}OT_{34}|j,m,01\rangle &\to (\mathbb{1}_2\otimes\hat{S}^1_{4,+})(\mathbb{1}_2\otimes\hat{S}^2_{4,+})\\
C\hat{N}OT_{34}|j,m,11\rangle &\to (\mathbb{1}_2\otimes\hat{S}^1_{4,-})(\mathbb{1}_2\otimes\hat{S}^2_{4,-})\\
C\hat{N}OT_{34}|j,m,10\rangle &\to (\mathbb{1}_2\otimes\hat{S}^1_{4,+})(\mathbb{1}_2\otimes\hat{S}^2_{4,+})
\end{aligned} \tag{22}$$

and

$$\begin{aligned}
C\hat{N}OT_{54}|j,m,00\rangle &\to \mathbb{1}\\
C\hat{N}OT_{54}|j,m,01\rangle &\to (\mathbb{1}_2\otimes\hat{S}^m_{4,+})\\
C\hat{N}OT_{54}|j,m,11\rangle &\to (\mathbb{1}_2\otimes\hat{S}^m_{4,-})\\
C\hat{N}OT_{54}|j,m,10\rangle &\to \mathbb{1}
\end{aligned} \tag{23}$$

Similarly, by having another combinations of the shift operators $\hat{S}_{2,\pm}$ and $\hat{S}_{4,\pm}$ Eq. (1) and coin operators Eq. (2), one can easily implement the CNOT operation on fifth qubit as both target $CNOT_{i5}$ or control $CNOT_{5i}$ here, $i-$ is the control or target qubit, respectively.

Universal computation on the $(n-1)^{\text{th}}$ and n^{th} qubit of the n-qubit system using quantum walk scheme when n is odd number, will require $(n-1)/2$ sets of quantum walk with four position basis states. The walk is defined on combined Hilbert space $\mathcal{H}_c\otimes\mathcal{H}_4\otimes...\otimes\mathcal{H}_4$ as shown in Fig. 1b of the main manuscript. Similarly, if n is even in n-qubit system, it will require $(n/2)-1$ sets of quantum walk with four position basis states and one set of quantum walk with two position basis states. Here the walk will be defined on Hilbert space $\mathcal{H}_c\otimes\mathcal{H}_4\otimes...\otimes\mathcal{H}_2$ as shown in Fig. 1a. $\mathcal{H}_c$ is the coin Hilbert space with two internal states $\{|0\rangle, |1\rangle\}$ which acts as the coin for the position Hilbert space $\mathcal{H}_4$ of the first set of the cyclic quantum walk with four computational basis states $\{|0\rangle, |1\rangle, |2\rangle, |3\rangle\}$ equivalent to $\{|00\rangle, |01\rangle, |11\rangle, |10\rangle\}$, respectively. The position space of the first set of cyclic quantum-walk will act as coin for the next set of quantum walk with four position basis states and so on. This scheme can be scaled to n- qubit system by using the position space of the previous set of quantum-walk as coin for the position space of next set of the quantum walk.

Table 2. Phase operation $\hat{P}$ on even $(n-1)^{\text{th}}$ and odd n^{th} qubit when the processor has n number of qubits. $|m\rangle$ is the position basis states of the previous set of cyclic quantum walk. $|(n-1)\rangle \otimes |n\rangle$ is $\{|00\rangle, |01\rangle, |11\rangle, |10\rangle\}$ which is also equivalent to the four computational position basis states of the cyclic quantum walk.

	$\hat{P}_{(n-1)}$	$\hat{P}_n$
$\|j,...,m,00\rangle$	$\mathbb{1}\|j,...,m,p=0\rangle$	$\mathbb{1}\|j,...,m,p=0\rangle$
$\|j,...,m,01\rangle$	$\mathbb{1}\|j,...,m,p=1\rangle$	$(\hat{\Phi}\otimes\mathbb{1})\|j,...,m,p=1\rangle$
$\|j,...,m,11\rangle$	$(\hat{\Phi}\otimes\mathbb{1})\|j,...,m,p=2\rangle$	$(\hat{\Phi}\otimes\mathbb{1})\|j,...,m,p=2\rangle$
$\|j,...,m,10\rangle$	$(\hat{\Phi}\otimes\mathbb{1})\|j,...,m,p=3\rangle$	$\mathbb{1}\|j,...,m,p=3\rangle$

Hadamard operation: Generalised scheme of quantum walk computation to implement Hadamard operation on $(n-1)^{\text{th}}$ and n^{th} qubit of the n-qubit system when n is odd is given in the table 1. An illustration of the scaling of the quantum walk scheme when the number of qubit in the system is odd is given in Fig. 1b of the main manuscript. Quantum walk scheme illustration is shown in Fig. 1a of the main manuscript when the number of qubit in the system is even and to implement Hadamard operation on the last qubit $|l\rangle$ when the number of qubits in the system is even is given by,

$$\begin{aligned}\hat{H}_l|j,...,m,0\rangle &= (\mathbb{1}\otimes\hat{V}_+^{(m \mod 4)})(\hat{H}_{p-1}\otimes\mathbb{1}_2)|j,...,m,0\rangle\\ \hat{H}_l|j,...,m,1\rangle &= (\mathbb{1}\otimes\hat{V}_-^{(m+1 \mod 4)})(\hat{H}_{p-1}\otimes\mathbb{1}_2)|j,...,m,1\rangle\end{aligned} \tag{24}$$

Phase operation: Phase operation on $(n-1)^{\text{th}}$ and n^{th} qubit of the n-qubit system when n is odd in number is given in the table 2 on quantum walk scheme. Similar to Hadamard operation, phase operation on the last qubit $|l\rangle$ when the number of qubit in the system is even is given by,

$$\begin{aligned}\hat{P}_l|j,...,m,0\rangle &= \mathbb{1}|j,...,m,0\rangle|j,...,m,0\rangle\\ \hat{P}_l|j,...,m,1\rangle &= (\hat{\Phi}\otimes\mathbb{1})|j,...,m,1\rangle\end{aligned} \tag{25}$$

An illustration of the scaling of the quantum walk scheme when the number of the qubit in the system is odd and even is shown in Figs. 1b and 1a, respectively in the main manuscript.
Controlled-NOT operation: CNOT operation can be implemented between any two qubits using quantum walk scheme with the help of the coin and shift operators given in the Eqs. (1) and (2) of the main manuscript along with identity operation in a similar way as CNOT operation has been shown for four and five qubit system. The quantum walk scheme will need same setup as shown in Figs. 1a and 1b of the main manuscript for a system with even and odd number of qubits.

S2 An illustration of 3-qubit Grover's Search Algorithm with a DTQW

An example of the quantum walk based search algorithm, on search space of a three-qubit system with the state $|011\rangle$ marked as our target state is presented below.

1. We start with a state $|\psi\rangle = \frac{1}{\sqrt{2}}\Big(|0\rangle_c + |1\rangle_c\Big)\otimes\frac{1}{\sqrt{4}}\Big(|00\rangle + |01\rangle_p + |10\rangle_p + |11\rangle_p\Big)$.

   Thus, we have,

$$\begin{aligned}|\psi\rangle &= \frac{1}{\sqrt{8}}\Big(|0\rangle_c|00\rangle_p + |0\rangle_c|01\rangle_p + |0\rangle_c|10\rangle_p + |0\rangle_c|11\rangle_p\\ &\qquad + |1\rangle_c|00\rangle_p + |1\rangle_c|01\rangle_p + |1\rangle_c|10\rangle_p + |1\rangle_c|11\rangle_p\Big)\\ &= \Bigg(\frac{\cos\frac{\theta}{2}}{\sqrt{7}}\Big(|0\rangle_c|00\rangle_p + |0\rangle_c|01\rangle_p + |0\rangle_c|10\rangle_p + |1\rangle_c|00\rangle_p\\ &\qquad + |1\rangle_c|01\rangle_p + |1\rangle_c|10\rangle_p + |1\rangle_c|11\rangle_p\Big) + \sin\frac{\theta}{2}\Big(|0\rangle_c|11\rangle_p\Big)\Bigg)\end{aligned} \tag{26}$$

   where $\cos\frac{\theta}{2} = \sqrt{\frac{7}{8}}$ and $\sin\frac{\theta}{2} = \sqrt{\frac{1}{8}}$.

2. Now we apply the oracle on this $|\psi\rangle$. The oracle for target state $|011\rangle$ is represented by the following operation

$$\hat{O} = \mathbb{1}\Big(|00\rangle_p\langle 00| + |10\rangle_p\langle 10| + |01\rangle_p\langle 01|\Big) + N_0\Big(|11\rangle_p\langle 11|\Big) \tag{27}$$

where the definition of $\hat{N}$ operators is given in Eq. 31 of the main manuscript. The above operation applies identity operator on $|00\rangle, |10\rangle, |01\rangle$ positions states and hence the probabilities in these position states are left untouched. On the position state $|11\rangle$, however, the effect of this operation may be seen as,

$$\begin{aligned}\hat{O}\,|0\rangle_c\,|11\rangle_p &= N_0\,|0\rangle_c\,|11\rangle_p \\ &= \hat{C}(0,0,\pi)\otimes \mathbb{1}\,|0\rangle_c\,|11\rangle_p \\ &= \begin{bmatrix}-1 & 0\\ 0 & 1\end{bmatrix}\begin{bmatrix}1\\0\end{bmatrix}\otimes \mathbb{1}\,|11\rangle_p \\ &= -\,|0\rangle_c\,|11\rangle_p\end{aligned} \tag{28}$$

$$\begin{aligned}\hat{O}\,|1\rangle_c\,|11\rangle_p &= N_0\,|1\rangle_c\,|11\rangle_p \\ &= \hat{C}(0,0,\pi)\otimes \mathbb{1}\,|1\rangle_c\,|11\rangle_p \\ &= \begin{bmatrix}-1 & 0\\ 0 & 1\end{bmatrix}\begin{bmatrix}0\\1\end{bmatrix}\otimes \mathbb{1}\,|11\rangle_p \\ &= |1\rangle_c\,|11\rangle_p\end{aligned} \tag{29}$$

So the final state after oracle operation is

$$\begin{aligned}|\psi'\rangle = \Bigg(\frac{\cos\frac{\theta}{2}}{\sqrt{7}}\Big(&|0\rangle_c|00\rangle_p + |0\rangle_c|01\rangle_p + |0\rangle_c|10\rangle_p + |1\rangle_c|00\rangle_p \\ &+ |1\rangle_c|01\rangle_p + |1\rangle_c|10\rangle_p + |1\rangle_c|11\rangle_p\Big) - \sin\frac{\theta}{2}\Big(|0\rangle_c|11\rangle_p\Big)\Bigg).\end{aligned} \tag{30}$$

3. Similar to step 2 above, the operation in Fig. 3 of the main manuscript gives all states except $|0\rangle_c|00\rangle_p$ a phase of π, and upon applying the Hadamard operation gives,

$$\begin{aligned}|\psi''\rangle = \Bigg(\frac{\cos\frac{3\theta}{2}}{\sqrt{7}}\Big(&|0\rangle_c|00\rangle_p + |0\rangle_c|01\rangle_p + |0\rangle_c|10\rangle_p + |1\rangle_c|00\rangle_p \\ &+ |1\rangle_c|01\rangle_p + |1\rangle_c|10\rangle_p + |1\rangle_c|11\rangle_p\Big) - \sin\frac{3\theta}{2}\Big(|0\rangle_c|11\rangle_p\Big)\Bigg).\end{aligned} \tag{31}$$

4. The Grover iteration (step 2 and 3) is then performed $\left\lceil\left(\frac{\arccos\sqrt{\frac{1}{8}}}{2\arccos\sqrt{\frac{7}{8}}}\right)\right\rceil = 2$ times and the final state is given by,

$$\begin{aligned}|\psi'''\rangle = \Bigg(\frac{\cos\frac{5\theta}{2}}{\sqrt{7}}\Big(&|0\rangle_c|00\rangle_p + |0\rangle_c|01\rangle_p + |0\rangle_c|10\rangle_p + |1\rangle_c|00\rangle_p \\ &+ |1\rangle_c|01\rangle_p + |1\rangle_c|10\rangle_p + |1\rangle_c|11\rangle_p\Big) - \sin\frac{5\theta}{2}\Big(|0\rangle_c|11\rangle_p\Big)\Bigg).\end{aligned} \tag{32}$$

The probability of obtaining the target state upon measuring the coin and position space qubits of the particle is then found to be $\left|\sin^2\left(\frac{5\theta}{2}\right)\right|^2 = 0.945$, as expected. One major advantage of this scheme is that it does not require ancilla qubits and both the oracle and iteration operations are just position dependent coin operations.

References

[1] Singh, S., Chawla, P., Sarkar, A. & Chandrashekar, C. M. Universal quantum computing using single-particle discrete-time quantum walk. *Scientific Reports* **11**, 11551, DOI: 10.1038/s41598-021-91033-5 (2021).
